# Increasing the strength of nanocrystalline steels by annealing: Is segregation necessary?

**DOI:** 10.1016/j.scriptamat.2014.09.023

**Published:** 2015-01-15

**Authors:** O. Renk, A. Hohenwarter, K. Eder, K.S. Kormout, J.M. Cairney, R. Pippan

**Affiliations:** a Erich-Schmid-Institute of Materials Science, Jahnstrasse 12, 8700 Leoben, Austria; b Department of Materials Physics, Montanuniversität Leoben, Jahnstrasse 12, 8700 Leoben, Austria; c Australian Centre for Microscopy and Microanalysis, The University of Sydney, Sydney, NSW 2006, Australia

**Keywords:** Nanocrystalline 316L, Hardening, Annealing, Segregation, Atom probe tomography

## Abstract

Hardening phenomena in nanocrystalline metals after annealing have been widely reported, and the subject of much recent debate. Solute segregation to grain boundaries and dislocation source hardening have been proposed to cause the strengthening. To shed light on the dominant mechanisms, we present results from mechanical experiments and atom probe tomography on samples with similar grain size but different amounts of solute segregation and different boundary chemistries.

  Since the pioneering work of Gleiter [1], nanocrystalline (nc) materials have received enormous attention in the past decades. It is well known that this class of material displays not only outstanding mechanical properties, such as high tensile strength or fatigue strength, but also enhanced physical properties, e.g. magnetic properties [2], [3]. The production of nc materials is not only restricted to bottom-up processes such as inert gas condensation or electrodeposition [1]; severe plastic deformation (SPD) methods such as high-pressure torsion (HPT) enable the synthesis of fully dense, bulk nc materials with grain sizes significantly smaller than 100 nm [4]. The advantage of SPD processing is that relatively large bulk samples can be processed, which allows mechanical tests to be performed that require a substantial sample volume, such as fracture toughness or fatigue testing [5], [6]. Although plasticity is still dislocation mediated in the grain size regime of ∼20–100 nm [7], [8], distinct changes are observed in the deformation characteristics when compared with coarse-grained metals. In this grain size regime, grain boundaries act as sinks and sources for dislocations [7], [8]. Characteristic dislocation cells, which build up in coarse-grained metals during plastic deformation or fatigue, have not been observed in ultrafine-grained or nc metals, because the grain size is smaller than (or at least of the same order as) these cells [9], [10]. Furthermore, unusual hardening phenomena after annealing treatments well below the temperatures at which grain growth occurs have been observed for nc materials produced by both SPD techniques and bottom-up processes such as electrodeposition (e.g. [11], [12], [13], [14]).

Although these hardening phenomena have been recognized for several years, the mechanisms behind them are still the subject of much debate in the literature. Some of the explanations are outlined here.

For pure nc materials, hardening has been attributed to a significant decrease in the dislocation density within the grains and a relaxation of the boundary structure during heat treatment. The large number of dislocation sinks (grain boundaries) present during annealing may cause a significant reduction in the dislocation density. As a consequence activation of dislocation sources is needed to realize plastic strain after the heat treatment [13], [15]. Similar results were found in MD simulations [16]. The bowing out of a dislocation from a grain boundary that has rearranged into a more equilibrium-like structure is expected to be rather difficult, necessitating high *s*tress levels for the movement of dislocations after annealing [13], [15], [16]. Interestingly, similar results were obtained during cyclic deformation of nc materials. Experiments and molecular dynamic (MD) simulations showed that plastic strain can cause a similar change in the grain boundary structure to that observed after annealing, leading to cyclic hardening of the samples [17], [18]. Similar behaviour was also observed during cyclic loading of the nc 316L steel used in the present study [19].

Other groups explain the hardening phenomenon on the basis of small amounts of impurity atoms that segregate to the boundaries of the nanocrystallites during annealing. Their contribution to the hardening remains unclear [15]. Segregated solute might supress dislocation emission from the boundaries, pin them or hinder relaxation of the dislocation at the boundaries, all leading to higher stress levels for plastic deformation of the material [15], [20], [21]. Indeed, the hardening behaviour upon annealing was often attributed to segregated solute for both alloys and metals that contain a certain amount of impurities [22], [23]. Interestingly, the unexpected strength increase in 316L steel was attributed to segregated solute too [24]. In addition, recent MD simulations indicated that solute segregation should lead to enormous strengthening effects [25].

In this paper we present a carefully designed combination of mechanical and microstructural characterization by atom probe tomography (APT) to clarify the question of whether solute segregation or second-phase particles at the boundary can account for the hardening behaviour observed. Annealing treatments of a nc 316L austenitic steel allowed variation of the structure and chemistry of the interface without changing the grain size. By following this methodology, structures with different amounts of solute segregated to the boundaries can be tested mechanically. Linking APT results and the mechanical datasets should clarify whether segregation is necessary for the hardening observed.

Disks of 316L austenitic stainless steel (max. 0.03 C, 0.30 Si, 1.70 Mn, 17.50 Cr, 14.50 Ni, 2.70 Mo, all values in wt.%), 35 mm in diameter and 7.5 mm in height, were severely deformed by quasi-constrained HPT for 15 revolutions at a constant rotation speed of 0.07 rpm with an applied pressure of 3.60 GPa at room temperature, resulting in an equivalent strain ε = 116 at a radius of *r *= 16 mm. Details of the setup used and the HPT process itself can be found elsewhere [4], [26]. The HPT process leads to a significant grain refinement down to grain sizes of ∼50 nm, investigated by transmission electron microscopy (TEM) and reported in Refs. [19], [27]. To modify the structure as well as the chemistry of the boundaries, isochronal (0.5 h) and isothermal heat treatments at 823 K were subsequently carried out on the HPT-processed material. Microhardness measurements were conducted on both the annealed and the as-deformed samples to test the influence of the annealing treatments on the mechanical properties. Microhardness measurements of the isochronally (0.5 h) annealed samples in the temperature range of 573–973 K are shown in Figure 1a. Figure 1a shows that the hardness increased steadily with increasing annealing temperature up to temperatures of 823 K (∼0.5T_m_). In fact hardness increased by 20% from 5 GPa for the as-HPT-processed material up to 6.10 GPa after the 0.5 h annealing treatment at 823 K. Additionally the hardness values of the nc material are compared with heavily cold-rolled (130% logarithmic thickness reduction) coarse-grained material in Figure 1a. For annealing temperatures higher than 823 K the hardness dropped, which can be attributed to partial grain growth of the structure while parts of the material still remained in the nc state [19]. TEM observations of the material annealed at 823 K showed no evidence of thermally induced grain growth (see Fig. 2). The grain boundaries of the annealed material (Fig. 2b) appear sharper when compared to the as-deformed condition (Fig. 2a), which can be attributed to relaxation of grain boundaries and internal stresses. This is confirmed by a decrease in the full width half maximum (FWHM) values of the {1 1 1} peaks obtained by X-ray diffraction by 30% upon annealing. As the grain size is not changing, this is only possible if defects are annealing out.

**Fig. 1:**
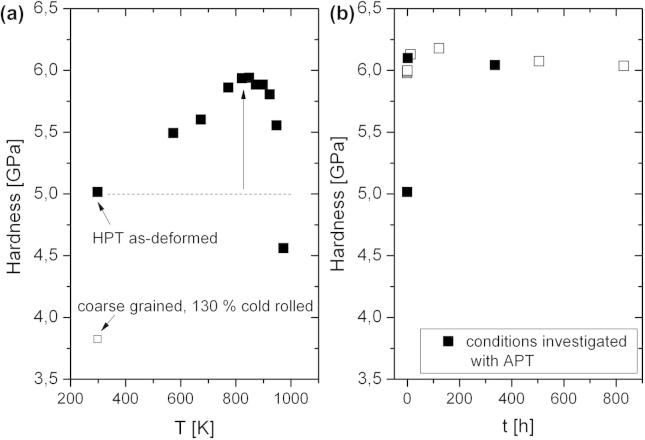
(a) Hardness of an isochronally (30 min) annealed nc austenitic steel as a function of the annealing temperature. The maximum hardness increase ΔH_max_ at 823 K is indicated with an arrow. The hardness value of coarse-grained heavily cold-rolled material is given for comparison. (b) Hardness of the nc austenite annealed at 823 K as a function of annealing time.

**Fig. 2:**
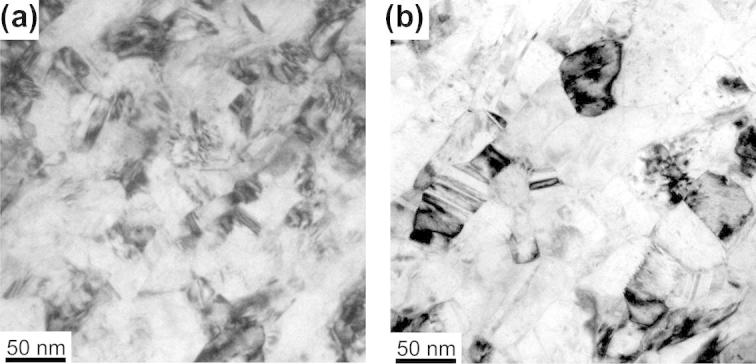
TEM bright-field images of 316L steel in various conditions: (a) as HPT deformed; (b) HPT deformed + 30 min/823 K.

To study the kinetics of the hardening process, isothermal annealing treatments at 823 K were carried out for different time intervals. The results of these heat treatments on the microhardness are presented in Figure 1b. It can be seen that the hardening process takes place rapidly and the maximum hardness value of 620 HV was achieved after 2 h of annealing. Slightly lower values of 610 HV were achieved after 5 min. No change in the mechanical properties was observed, even after annealing the material for 830 h, indicating an unprecedented thermal stability of the nc austenitic steel.

To link the mechanical data with possible changes in the grain boundary chemistry due to solute segregation, APT was carried out on as-deformed samples, samples annealed for a short time (1.5 h) as well as samples annealed for a long time (325 h). For specimen preparation, the samples were dissected into 0.6 mm × 0.6 mm square rods, which were then sharpened via electropolishing to create needles. Rough polishing was conducted with an electrolyte of 25% perchloric acid in (70%) glacial acetic acid and a voltage of between 10 and 18 V DC. Fine polishing was performed in 2% perchloric acid in 2-butoxyethanol at a voltage of 13–18 V DC. The atom probe experiments were carried out on a Cameca LEAP 4000X Si in laser pulsing mode at a specimen temperature of 25 K, a pulse rate of 500 Hz, a pulse energy between 100 and 170 pJ and a target evaporation rate of 1.5%. IVAS 3.6.4 software was used for the reconstruction and visualization of the APT data.

Grain boundaries in the obtained datasets were identified and the Gibbsian interfacial excess of solute (Γ) calculated. Details of the methods for the calculation of Γ can be found in Refs. [28], [29]. Figure 3 shows the interfacial excess of Si (Γ_Si_) of the as-deformed specimen (displayed in red) and the specimen annealed for 1.5 h at 823 K (displayed in blue). The graph shows the average interfacial excess along 7 selected regions of grain boundaries for the as-deformed specimen and 37 regions for the annealed specimen (each region was a cylindrical volume of data 20 nm in length and 10 nm in diameter). The interfacial excess varied widely, and the error bars show the standard deviation of these measurements. The inset images are regions of datasets obtained showing representative volumes used to determine the interfacial excess values. 14% of the Si atoms are displayed. The results show very little excess Si on the grain boundaries of the as-deformed specimen. This indicates that, for an austenitic steel, HPT does not lead to deformation-induced segregation as reported previously for several Al alloys [20], [30]. This might be explained by the considerably lower homologous deformation temperature of the austenitic steel. However, segregation can also be diminished again by heavy plastic deformation, as shown for cold-drawn pearlitic steel wires [31].

**Fig. 3:**
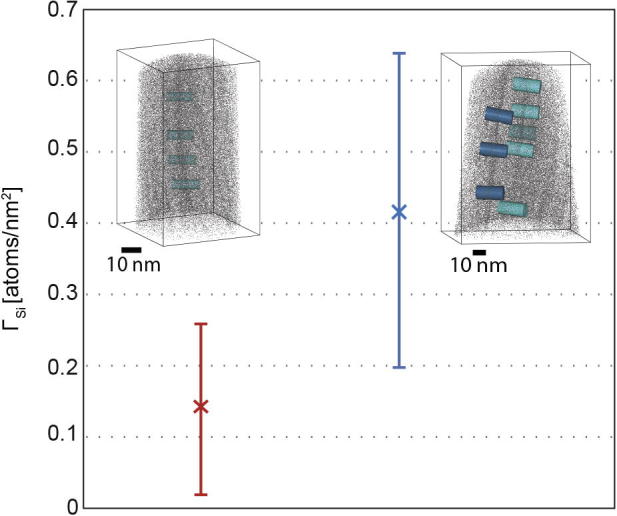
Interfacial excess of Si (Γ_Si_) of the as-deformed specimen (red) and the short-term annealed specimen (1.5 h) (blue), with small images of APT reconstructions for each specimen.

The samples annealed for 1.5 h showed Γ_Si_ values in the range of 0.2–0.64 Si nm^−2^, indicating significant segregation of Si towards the grain boundaries. The variation of the Γ_Si_ values arises due to inhomogeneous segregation over the length of the grain boundaries. The grain boundaries of the APT datasets were also checked for segregation of all other elements in the sample, but apart from Si, only traces of Mo, Cr and Ni segregated to the boundary.

After 325 h of annealing at 823 K, in addition to increased Si segregation, Mo and Cr segregated to the boundaries. In these specimens, Mo and Si formed second-phase particles of ∼5–10 nm in thickness and up to 90 nm in length. This can be seen in the APT reconstruction shown in Figure 4a and b. A proxigram [32], [33] showing the composition of all second-phase particles, together with segregation of Cr in the vicinity of the matrix–particle interface, is provided in Figure 4c. This plot shows the average composition moving away from an isoconcentration surface (here 14.24% Mo) in both directions, providing a concentration profile that accounts for the 3-D nature of the grain boundaries. This approach averages any effects from different levels of segregation to different interfaces, as well as any effects that arise from trajectory aberrations in atom probe data when the boundaries are oriented differently with respect to the analysis direction. The proxigram reveals that the second-phase particles contain about 37 at.% Fe, 25 at.% Mo, 10 at.% Si, 23 at.% Cr, 2 at.% Ni, 1 at.% Mn and 0.5 at.% C. In comparison, the matrix consists of 71 at.% Fe, 0.7 at.% Mo, 1 at.% Si, 20 at.% Cr, 5 at.% Ni, 2 at.% Mn and 0.01 at.% C. Figure 4b shows a slice of the APT dataset shown in Figure 4a, taken in the z direction. Figure 4d is a 1-D concentration profile taken from the cylindrical region of interest shown in Figure 4b. This profile was taken perpendicular to a second-phase particle. Consistent with the proxigram analysis, it shows that the Cr, Mo, Si and C partition to the second-phase particle, and the particles are lean in Fe, Ni and Mn. Beside the particles, additional Si excess Γ_Si_ = 0.36 Si nm^−2^ was found at the boundary. However, apart from the particles, no Mo, Cr and Ni was found for the 325 h annealed samples which is different to the samples annealed for a short time.

**Fig. 4:**
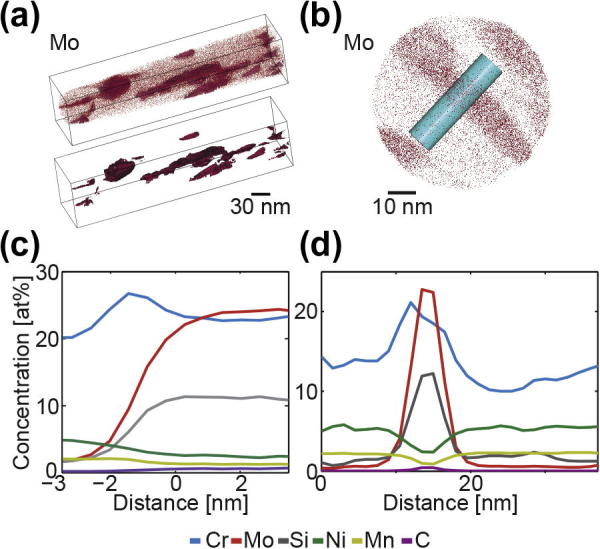
(a) APT reconstruction for Mo of the long-term annealed sample (325 h). The second APT reconstruction below shows the Mo isoconcentration surfaces of the second-phase particles. (b) Slice of the APT dataset in the z direction with a region of interest (turquoise cylinder) perpendicular to a second-phase particle. (c) Average proxigram from all Mo isoconcentration surfaces (second-phase particles). (d) 1-D concentration profile along the z axis of the of the turquoise cylinder shown in (b).

Although the annealing temperature of 823 K would be adequate for precipitation of σ-phase, no indication of such phases was found by TEM and transmission Kikuchi diffraction. Additionally the chemical composition of the particles is far from that reported for σ-phase in 316L steel [34]. This further indicates that 316L steel remains fully austenitic also during SPD.

The relatively large amount of Si and Mo in the second-phase particles may be explained by the fact that Mo and Si are known to stabilize the ferritic phase. It is likely that these particles would grow further by Ostwald ripening during longer annealing times until the number of particles is too small to stabilize the nc structure against growth. The excellent thermal stability of the nc austenitic steel at 0.5T_m_ can be directly attributed to the excess of solute, lowering the grain boundary energy, as treated theoretically in Ref. [35]. For longer annealing times second-phase particles are formed and stabilize the grain boundary, which is sometimes referred as kinetic stabilization [36].

Despite large modifications of the grain boundary chemistry, increasing amounts of Si and Mo segregated to the grain boundary, and finally the formation of second-phase particles after long annealing times, no changes in the mechanical properties have been observed. If solute strongly influenced the mechanical behaviour, distinct hardness changes would be noticed for the samples annealed for different annealing times. Thus linking APT results and mechanical properties, it can be concluded that the mechanical behaviour of this nc austenitic steel is independent of the amount of solute present at the boundaries. Furthermore the hardening takes place rapidly: after only 5 min of annealing at 823 K the hardness reached values of ∼95% of the peak hardness. APT revealed only slight Si decoration even after 1.5 h of annealing at the same temperature, and thus the hardening results are thought to be mainly the result of a dislocation starvation process.

The measured hardening effect during annealing can therefore be attributed to the annihilation of relatively mobile dislocations and a relaxation of the grain boundaries, making emission of dislocations or their relaxation at the boundaries difficult. A strong indication for this explanation is the early strain localization during testing of annealed specimens, as the samples will undergo pronounced strain softening, leading to a significantly reduced ductility [13], [15].

Despite these observations, solute segregation is a prerequisite for the observed hardening phenomena but not the actual origin. Segregated solute stabilize the nanocrystallites against grain growth during annealing while competitive processes such as dislocation annihilation and boundary relaxation prevail. These processes, which are thought to be the source for the hardness increase, remain widely unaffected by the boundary chemistry.

To conclude, severely plastically deformed 316L austenitic steel showed an unprecedented increase in hardness by 20% upon annealing. Combining mechanical data and results from APT revealed that this phenomenon is apparently not related to solute segregation and definitely not to second-phase particles at the boundary. Nevertheless, solute or second-phase particles stabilize the nc structure and therefore allow for the annihilation and relaxation processes necessary for the hardening phenomena during annealing.
